# A Comparison Between the Effect of Cuminum Cyminum and Vitamin E on the Level of Leptin, Paraoxonase 1, HbA1c and Oxidized LDL in Diabetic Patients

**Published:** 2017-01-24

**Authors:** Ghatreh Samani Keihan, Mohammad Hossein Gharib, Ali Momeni, Zohreh Hemati, Roya Sedighin

**Affiliations:** 1 *1.Clinical Biochemistry Research Center, Shahrekord University of Medical Sciences, Shahrekord, Iran.*; 2 *Department of Internal Medicine, Shahrekord University of Medical Sciences, Shahrekord, Iran.*; 3 *Shahrekord University of Medical Sciences, Shahrekord, Iran.*

**Keywords:** Diabetes, vitamin E, cumin, leptin, paraoxonase1, oxidized LDL

## Abstract

Diabetes is one of the most common metabolic diseases in the world. Vitamin E reduces protein glycation and improves insulin sensitivity, while cumin is effective in remission of diabetes. Therefore this study was designed to evaluate the effects of vitamin E and cumin essential oil, on the blood level of leptin,glycosylated hemoglobin (HbA1C) and also on lipid profile in diabetic patients.In this double blind clinical trial, 95 diabetic patients were selected and randomly dividedinto three groups.The first group received cumin essential oil in capsule form. The second group received Vitamin E, and the third group was used ascontrol receiving oral gelatin capsules as placebo for three months period.Blood glucose, lipid profile, apolipoprotein A1 (ApoA1), apolipoprotein B (ApoB), leptin, HbA1c, oxidized LDL (oxLDL), and paraoxonase1 activity were measured. The results showed reduction in oxLDL and significant increase in paraoxonase 1 in Vitamin E group by the end of the third month period (P<0.05). Cumin group showed decrease in blood glucose, HbA1C, triglyceride, leptin and ox-LDL. ApoA1 and paraoxonase1 were also increased by cumin treatment (P<0.05).Diabetic complications may have been reduced by intake of Vitamin E and cumin essential oil. Cumin in comparison with vitamin E has broader impact and it is more beneficial in terms of ability to reduce the diabetic index.

Diabetes is one of the most common metabolic diseases in the world,and several factors are involved in its pathogenesis (1). Glycosylated hemoglobin (HbA1c) is formed by an irreversible, nonenzymatic binding of glucose to hemog-lobin.HbA1c is a marker of metabolic regulation in diabetic patients. Incomplete metabolic regulation in diabetes is the leading cause of various complications,giving rise to oxidizing compounds (2). Leptin is a polypeptide hormone secreted by adipose tissue and involved in the control of body weight (3). Insulin is an important regulator for leptin and could have an impact onthe expression of the gene encoding leptin and also on the corresponding receptor (4). Vitamin E reduces the oxidation of lipoproteins, oxidative damage of the endothelium, proteins glycation, and consequently improves insulin sensitivity (5). Some studies have shown that low levels of vitamin E are directly associated with diabetes, causing diabetes compli-cations (6). There are contradictory data on the effect of vitamin E on insulin and blood sugar (7).

Cumin is an herbaceous aromatic plant, yearlong; in the Apiaceae family with the scientific name of "cuminum cyminum". Cumin is harvested in Iran and it is used in traditional medicine, drinks flavor and desserts, and also as a fragrant compo-nent in creams, lotions and perfumes.Cumin is used in the treatment of various diseases and is effective in remission of diabetes. The fruit of cumin contains 2-5% essential oil which is mainly made of paracetamol, alpha and beta pinene, propanal, cumin alcohol, cuminaldehyde, alpha terpineol and myrcene (8).

In a study comparing the anti-diabetic efficacy of cumin and glibenclamide, cumin effects in preventing the free radical formation, decreasing glucose and protein glycation end products was very much improved in comparison with gliben-clamide (9). In another study, the impact of cumin on blood glucose and oxidized LDL (oxLDL) decrease, and increase in arylesterase activity of paraoxonase 1 was reported (10).

Increased activity of paraoxonase 1 induces an increase in the concentration of HDL in serum, confirming that increased paraoxonase 1 activity leads to less cardiovascular disease in humans (11). The present study was designed to evaluate the effect of vitamin E and cumin essential oil, on blood leptin, HbA1c and lipid profile of diabetic patients.

## Materials and methods

**Patient selection and sampling**

This study was approved by the Medical Research Ethics Committee of Shahrekord University of Medical Sciences and was recorded in the registry of clinical trials Iran with IRCT2014100619420N1 code. Consent forms and the questio-nnaires were signed and filled in by all patients.

In this double blind clinical trial study, 105 diabetic patients were recruited. Patients with at least 3 years disease history were referred by an internal medicine physicians after interview and patient’s consent. All patients were under treatment with metformin as antidiabetic drug.

Inclusion criteria were as follows: patients with type 2 diabetes without obvious complication who were aged 30-75 years) 7(, while smokers, subjects with the underlying disease such as cardiovascular disease and other metabolic disorders like hypercholesterolemia, alcohol, supplements and drug users, were excluded from the study. Ten patients including 6 and 3 subjects from cumin and vitamin E groups respectively, and also one subject from control group, withdrew during the study.The laboratory data as well as questionnaires data were collected for analysis.

**Study design**

The patients were randomly assigned into 3 groups. The first group received cumin essential oil(in capsules resembling placebo and vitamin E capsules), the second group received vitamin E and the third group served as control and received oral gelatin capsules as placebo for a period of 3 months. Dosage of vitamin E was based on a similar study (12) and after approval by the physician, 800 IU (150 mg) was selected. Cumin dosage was determined as 25 mg/day according to the manufacturer's instructions. At the beginning of the study and after 90 days of intake of supplements in each group, 10 ml venous blood in fasting state was collected from all participants. Blood serum were stored at -20C before measuring leptin, lipid profile,paraoxonase1,levels. Whole blood containing EDTA was used HbA1c level measurement.

**Biochemical analyzes**

Blood glucose and lipid profile were measured using the autoanalyzer (BT-3000- Italy) and appropriate commercial kits from Pars Azmoon, Iran. Apolipoprotein A1 (ApoA1) and apolipo-protein B (ApoB) were measured by immuno-turbidimetry.Serum levels of leptin and oxLDL were detected by ELISA kit (Mercodia, Sweden). HbA1c was measured using column chromato-graphy with Nycocard Reader II.

Arylesterase activity of paraoxonase 1 was measured using phenylacetate as the substrate, by spectrophotometric method (11).

**Statistical analyzes**

The statistical analysis were conducted by SPSS software (version 18) using repeated measures ANOVA and independent t-test. P value <0.05 was considered as statistically significant.

## Results

The demographic characteristics and age distribution of the study population are shown in table 1. There was no significant difference between the 3 groups in body mass index, sex, age and blood pressure.

Biochemical data are summarized in table 2.

As shown in this table, the results indicate adecrease in oxLDL and a significant increase in paraoxonase 1 in Vitamin E group by the end of the third month (P<0.05). Results in cumin group showed a decrease in blood glucose, HbA1C, triglyceride, leptin and ox-LDL. Apo A1 and paraoxonase1 were increased after three months cumin intake (P<0.05).

## Discussion

In this study, some biochemical parameters were measured after three months of vitamin E and cumin essential oil intake by diabetic patients. The data before and after intake were compared.

Reduced level of oxLDL after vitamin E intake, is probably due to the antioxidant features of vitamin E and protective role of this vitamin in lipid oxidation,such that by reducing the quantity of oxidants, oxLDL is also reduced.

Following vitamin E intake, paraoxonase 1 enzyme activity has been increased in blood, showing that decrease in oxidative stress and oxidized lipids leads to higher enzyme activity. Our data suggest a specific relation between the increased enzyme activity and a decrease in lipid oxidation.

**Table 1:** The demographic characteristics

	**Group (N)**	**Mean**	± **SD**	**P-value**
Age (years)	Vit E (32) Cumin (29) Control (34)	57 59 61	6.88 6.9 6.56	0.069
Weight (kg)	Vit E (32) Cumin (29) Control (34)	77.8 80 73.8	11.9 16.5 9.30	0.338
BMI (kg/m^2^)	Vit E (32) Cumin (29) Control (34)	28.59 29.4 29.43	4.18 5.80 4.02	0.852
Systolic bp (mmHg)	Vit E (32) Cumin (29) Control (34)	138 140 139	12.2 8.10 18.2	0.952
Diastolic bp (mmHg)	Vit E (32) Cumin (29) Control (34)	76.5 72 76.88	8.83 7.53 10.9	0.657

Data as mean± SD; N= patients per group

**Table 2:** A comparison of Biochemical variables before and after the study

**Groups (N=105)**	**Cumin (29)**			**Vit E (32)**					**Control (34)**					
**Variables**	**Before-**	**After-**	**P value**	**Before**	**After**		**P value**		**Before**		**After**		**P ** **value**	
HbA1c (%)	8.55±0.4	7.35±0.21	0.003	7.99±0.4	8.53±0.4	0.164		8.68±0.3		9.08±0.3		0.12	
Glucose (mg/dl)	144.9±7.9	116.4±3.7	0.001	157.4±19	150.3±20	0.506		160±11.2		181±11.3		0.037	
Triglyceride (mg/dl)	204.3±32	158.6±22	0.050	364±441	254±231	0.065		264.2±37		288±38		0.237	
Cholesterol (mg/dl)	136.2±5	137.3±5	0.724	143.3±9.2	143.3±7.6	0.970		145.5±6.9		150.7±6.7		0.136	
Uric. Acid (mg/dl)	4.96±0.3	4.93±0.3	0.838	4.4±0.3	4.5±0.2	0.475		5±0.3		5±0.3		0.644	
Leptin (µg/ml)	26.38±8.3	20.2±5.8	0.008	24.9±7.6	24.8±6.2	0.971		24.7±5.1		33.6±6.5		0.023	
HDL (mg/dl)	35.3±2.2	38.7±2.3	0.087	38±3.7	37.8±2.2	0.938		34.3±2.1		32.4±2.7		0.215	
oxidized-LDL (U/L)	100.7±4.5	90.3±3.9	0.000	102.9±3.6	89.3±3.5	0.000		100.5±4.6		102.4±4.4		0.646	
Paraoxonase1 (U/L)	65.3±6.7	83.3±7.8	0.017	69.1±7.7	90.3±10.5	0.046		67.7±7.2		69.3±6.3		0.632	
ApoA1 (mg/dl)	102.9±4.6	115.4±4.5	0.014	103.6±5.3	110.5±4.7	0.098		102.3±5.7		97.7±5.2		0.241	
Apo B (mg/dl)	103.1±5.5	98.6±4.2	0.347	104.2±6.1	99.6±5.1	0.329		98.7±7.2		115.8±7.2		0.153	
LDL (mg/dl)	69.8±4.4	71.8±5.2	0.561	61.9±8.7	66.6±8.3	0.119		73.1±7.7		77.9±7.3		0.265	

Data presented as mean± SD; N= 30 per group. HbA1c (A1 glycated hemoglobin), HDL (high-density lipoprotein), LDL (low density lipoprotein), ApoA1 (apolipoprotein A1), Apo B (apolipoprotein B).

Shinde et al. have performed a study on 120 type 2 diabetic patients after three months vitamin E intake, showing a decrease in oxidative stress and an increase in antioxidant enzymes activity such as paraoxonase1 (13). But in contrast to the present study, Sarandolet al. showed that after three months vitamin E intake, no changes occurred in the activity of either the paraoxonase1or ApoB (14).

A study by Reaven et al. showed that 1600 IU vitamin E intake per day in men aged 50 to 70 years and having type 2 diabetes mellitus, led to LDL oxidation decrease in comparison with the control group (15).

Paraoxonase1 activity increase and oxLDL level decrease observed in the present study are consistent with most of the findings of the above mentioned studies. Obviously, these changes were anticipated based on the antioxidant characteristics of vitamin E.The effects of cumin on oxLDL level and paraoxonase1 activity were similar to vitamin E.

There are contradictory ideas about the effect of vitamin E on glycemic control. Gazes et al.showed that HbA1c was significantly reduced in type 2 diabetic patients, after 1600 IU a day intake of alpha-tocopherolfor a period of 8 weeks (16), but our data did not confirm this finding. The discrepancy can be due to the difference in the vitamin dosage used which was more than double in comparison with the present study. The other factor which may affect the data is the difference in the patient’s diet which was not under our control. Also, the medication used for lowering patient’s blood sugar was different among the subjects which may have an impact on the outcome of data. 

In a study by Paolisso et al., where type 2 diabetics received 900 mg/day alpha-tocopherol for a period of three months, the level of HbA1c was significantly reduced (17). However, in the study of Ble-castillo' et al., oral intake of α-tocopherol 800 IU/day by overweight female subjects, no change in blood glucose was observed, but HbA1c was markedly reduced (18). Relatively, the results of the present study have shown that vitamin E has no clear effect on HbA1c level.

Merzouk et al. showed that vitamin E intake reduced the total cholesterol, triglycerides, LDL and ApoB levels (19) whilst in a study by Cinaz et al., no significant difference was found between serum levels of cholesterol and triglycerides (20). In the present study, the level of HDL and cholesterol showed no changes but triglycerides level was slightly reduced. It is possible that more noticeable changes would be anticipated by extended use or an increase in vitamin E dosage.

Because the intake of vitamin E has no effect on the sugar, no significant changes in HbA1c has been expected. We speculate that vitamin E has no effect in the control of dyslipidemia as no significant changes have been observed in the level of cholesterol, triglycerides, HDL, the mass of fat cells and fat tissue leptin level, after this supplement intake. 

In a study by Ben et al., performed on 30 patients with type 2 diabetes, it was shown that vitamin E can have a negative regulatory effect on leptin secretion (21). Chappell et al. have evaluated the effect of antioxidants such as vitamin E on pregnant women and indicated that leptin was reduced by vitamin E intake (22), which has not been observed in our study. In a study conducted on pigs by Kükner et al., there was no difference between the leptin expression in the control group and the group treated with vitamin E (23). Similarly, in the present study Vitamin E did not have any effect on leptin. We speculate that genetic factors or different intake dosage of vitamin E can be the determining factors in such difference.

The present study showed that cumin essential oil intake for a period of three months caused a significant decrease in blood glucose, HbA1c, triglycerides, leptin, ox-LDL and a significant increase in paraoxonase 1 and ApoA1 concentra-tion.

Reduction of ox-LDL can be caused by antioxidant properties of cuminum cyminum (24) and the presence of flavonoids in its composition (25). Therefore, we anticipate that increasing the amount of paraoxonase 1 as an effective antioxidant enzyme may lead to the lipid oxidation reduction. Iron, zinc and manganese are among the compo-nents of the cuminum cyminum (26).

So it is possible that the presence of manganese in the structure of cumin helps the antioxidant enzymes such as manganese containing superoxide dismutase, to reduce the ox-LDL level.

In addition to the anti-oxidant property, cumin may have a specific effect in increasing HDL and lowering blood cholesterol, by increasing Apo A1.

Although the increase in ApoA1 has not been observed in some studies (10), but increased level of paraoxonase1 and decreased level of ox-LDL are consistent with the findings of the present study.

Some studies have reported a decrease in blood sugar, triglycerides, cholesterol and increase of HDL following cumin intake (27, 28), although another research suggested that cumin has no effect on lowering the cholesterol level (29). We observed no changes in cholesterol levels, but confirmed a decrease in blood sugar and trigly-cerides levels.

Cumin intake was the cause of blood glucose decrease in some of the above studies.This effect may be due to high levels of manganese and zinc in cumin. It was demonstrated that manganese and zinc are involved in the production and secretion of insulin, insulin sensitivity and carbohydrate metabolism (30). The specific action of cumin may be due to the presence of cuminaldehyde which inhibits aldose reductase and alpha-glucosidase, as two enzymes which can help diabetes progression (9).

Three months after cumin essence intake, a reduction inleptin as white adipose mass index was observed which is consistent with the cumin anti-dyslipidemic effects. Due to the fact that cumin has a direct effect on insulin and leptin and both of them have an inhibitory effect on appetite and neuropeptide Y, it is likely that cumin has imposed its effect on these two hormones through the intervention in hypothalamic signals. On the other hand with regards to the effect of cumin on induction of insulin (31), this mechanism can be imposed indirectly on leptin through insulin.

Although not many studies have been performed yet on the relationship between leptin and cumin intake, the results of this study are consistent with other studies findings.

The difference in our data in comparison with other studies may be due to several factors such as type of vitamin E, type of cumin essence, subjects’ genetic background or duration of intake or inadequate dose used in our study.

In addition, in numerous studies each patient has used different medications for treatment of diabetes which can have an impact on the results outcome. On the other hand, diet type which certainly has an impact on the present study and data outcome, was not under our control.

The present study showed that improvement of lipid profile was more efficient with cumin intake in comparison with vitamin E.Cumin has been shown to reduce LDL oxidation by antioxidant activity and improve HDL composition by increasing Apo A levels.Therefore, cumin can be more effective than vitamin E in the prevention of cardiovascular complications in diabetic patients.

Cumin was found to have a better hypoglycemic effect than vitamin E. According to the present study, cumin can help control diabetes mellitus complications while this feature is not seen with vitamin E.The results of the present study may provide additional information on the control of diabetes complications and could help in reducing the damage of glycated proteins in diabetic patients. Also, vitamin E may reduce some complications in diabetic patients. Obviously, cumin in comparison with vitamin Ecan reduce more effectively the complications of diabetes.
